# Adjunctive transcranial alternating current stimulation for patients with major depressive disorder: A systematic review and meta-analysis

**DOI:** 10.3389/fpsyt.2023.1154354

**Published:** 2023-03-22

**Authors:** Wei Zheng, Dong-Bin Cai, Sha Nie, Jian-Hua Chen, Xing-Bing Huang, Stephan Goerigk, Andre Russowsky Brunoni, Wei Zheng

**Affiliations:** ^1^Xiamen Xian Yue Hospital, Xiamen, China; ^2^Shenzhen Traditional Chinese Medicine Hospital, Shenzhen, China; ^3^The Affiliated Brain Hospital of Guangzhou Medical University, Guangzhou, China; ^4^Department of Psychiatry and Psychotherapy, Ludwig-Maximilians-University, Munich, Germany; ^5^Department of Psychological Methodology and Assessment, Ludwig-Maximilians-University, Munich, Germany; ^6^Department of Psychology, Charlotte Fresenius Hochschule, Munich, Germany; ^7^Center for Clinical and Epidemiological Research and Interdisciplinary Center for Applied Neuromodulation, University Hospital, University of São Paulo, São Paulo, Brazil; ^8^Service of Interdisciplinary Neuromodulation, Department and Institute of Psychiatry, University of São Paulo Medical School, São Paulo, Brazil; ^9^Laboratory of Neuroscience and National Institute of Biomarkers in Psychiatry, Department and Institute of Psychiatry, University of São Paulo Medical School, São Paulo, Brazil

**Keywords:** major depressive disorder, transcranial alternating current stimulation (tACS), response, remission, meta-analysis

## Abstract

**Objective:**

We performed a meta-analysis of randomized, double-blind, controlled trials (RCTs) to systematically investigate the therapeutic effects and tolerability of transcranial alternating current stimulation (tACS) for the treatment of patients with major depressive disorder (MDD).

**Methods:**

Electronic search of PubMed, PsycINFO, EMBASE, Chinese National Knowledge Infrastructure, Wanfang database, and the Cochrane Library up to 1 April 2022. Double-blind RCTs examining the efficacy and safety of tACS for patients with MDD were included. The primary outcome was the improvement of depressive symptoms following a course of tACS treatment. Data were analyzed using Review Manager Version 5.3 (Cochrane IMS, Oxford, UK). Study quality was assessed using the Cochrane risk of bias and Jadad scale. Publication bias was assessed using a funnel plot and the Egger test.

**Results:**

We identified 883 articles, of which 4 RCTs with 5 active treatment arms covering 224 participants with MDD on active tACS (*n* = 117) and sham tACS (*n* = 107) were eligible for inclusion. Meta-analysis of depressive symptoms at post-tACS found an advantage of active tACS over sham tACS (*n* = 212, standard mean difference (SMD) = −1.14, 95% confidence interval (CI): −2.23, −0.06; *I*^2^ = 90%, *P* = 0.04). The significant superiority of active tACS over sham tACS in improving depressive symptoms remained in a sensitivity analysis. Active tACS was significantly superior to sham tACS regarding depressive symptoms at the 4 week follow-up (SMD = −1.07, 95% CI: −2.05, −0.08; *I*^2^ = 88%, *P* = 0.03) and study-defined remission [risk ratio (RR) = 2.07, 95% CI: 1.36, 3.14, *I*^2^ = 9%, *P* = 0.0006]. The discontinuation rate due to any reason was similar between the two groups (*P* > 0.05). All included studies were rated as high quality (Jadad score ≥ 3), with funnel plots of primary outcome not suggestive of publication bias.

**Conclusion:**

tACS appeared to be modestly effective and safe for improving depressive symptoms in patients with MDD, although further studies are warranted.

## Introduction

Major depressive disorder (MDD) is the common psychiatric illness, leading to the highest burden of disability among mental and substance use disorders (1). Despite antidepressants (ADs) and psychotherapies being available, many patients suffering from MDD do not adequately respond to ADs (2, 3) or psychotherapies (4). Consequently, augmentation strategies of ADs with non-pharmacological interventions, such as adjunctive non-invasive brain stimulation (NIBS) techniques (5), including transcranial magnetic stimulation (TMS), magnetic seizure therapy (MST), electroconvulsive therapy (ECT), transcranial direct current stimulation (tDCS), and transcranial alternating current stimulation (tACS), have been applied widely for the treatment of MDD in clinical practice.

As a type of NIBS technique, tACS is a newly developed intervention (6). In contrast to tDCS, which applies a constant current, tACS provides brain stimulation by sending alternating electric currents to the scalp (7). A case report found that gamma-tACS contributed to a good response in a patient with MDD during pregnancy, and the patient achieved remission during the 3 month follow-up (6). Although four recent randomized, double-blind, controlled trials (RCTs) (8–11) have examined the feasibility, efficacy and safety of tACS in treating adult patients with MDD, the findings have been inconsistent. Two RCTs consistently reported that tACS could significantly improve depressive symptoms in first-episode drug-naïve patients suffering from MDD (10, 11). However, in Alexander et al.’s (8) study, a negative finding was observed when comparing three different conditions (10 Hz-tACS, 40 Hz-tACS and sham tACS).

To date, no systematic review or meta-analysis on tACS as a therapeutic intervention for adult patients with MDD has been published. We hypothesized that active tACS would show superiority over sham tACS in reducing depressive symptoms of MDD.

## Materials and methods

### Eligibility criteria

The eligibility criteria of this systematic review used the *PICOS* framework. *P*articipants: adult patients (≥18 years) with MDD. *I*ntervention versus *C*omparison: active tACS versus sham tACS or active tACS plus ADs versus sham tACS plus ADs. *O*utcomes: The primary outcome was the improvement of depressive symptoms at post-tACS as measured by standardized rating scales, such as the Montgomery-Åsberg Depression Rating Scale (MADRS) and the Hamilton Depression Rating Scale (HAMD). As recommended previously (12, 13), HAMD was preferred to other scales when multiple rating scales (i.e., HAMD and MADRS) were used in the study. The secondary outcomes were (1) the improvement of depressive symptoms at the 2-week and 4-week follow-ups after tACS; (2) study-defined remission (i.e., HAMD total score ≤ 7) and response (i.e., reduction in HAMD total score ≥ 50%) at post-tACS; (3) adverse effects; and (4) any cause discontinuation. *S*tudy: Only published double-blind RCTs examining the efficacy and safety of tACS for the treatment of patients with MDD were included. Studies with a single-session of tACS were excluded.

### Search strategy

This meta-analysis was conducted and reported in line with the Preferred Reporting Items for Systematic Reviews and Meta-Analyses (PRISMA) statement (Supplementary Table 1) (14). Two authors (WZ and DBC) independently identified double-blind RCTs published prior to 1 April 2022, which examined the efficacy and safety of tACS for the treatment of MDD by systematically searching PubMed, PsycINFO, EMBASE, Chinese National Knowledge Infrastructure (CNKI), Wanfang databases, and the Cochrane Library. The search terms were as follows: (transcranial alternating current stimulation OR tACS) AND (depression OR depression OR depressive OR depressed OR melancholia). Furthermore, the reference lists of the identified articles (8–11) and relevant reviews (15, 16) were manually searched to avoid missing eligible studies. The more search details were presented in the Supplementary Text.

### Data extraction

Two authors (WZ and DB-C) independently extracted the following data from each included RCT using a tailored form: author, year of publication, sample size; number and percentage of male participants; average age of participants; illness duration; primary and secondary outcomes (efficacy, safety and tolerability of tACS for MDD). Any discrepancies were resolved by discussion and adjudication through a senior author. If the study data were unclear, the first/corresponding authors were contacted by email or telephone to obtain further information. As recommended previously (17), we used values from other studies to obtain an average when a study did not report the standard deviation.

### Data synthesis

Data synthesis was performed using Review Manager Version 5.3 (Cochrane IMS, Oxford, UK). The random-effects model was applied for all meta-analytic outcomes (18). For dichotomous and continuous outcomes, we calculated the risk ratio (RR) and standard mean difference (SMD) with 95% confidence intervals (CIs), respectively. Significant heterogeneity for the meta-analytic outcomes was defined as an *I*^2^ of > 50%. In the case of *I*^2^ > 50% for the improvement of depressive symptoms at post-tACS, a sensitivity analysis was performed to investigate the source of heterogeneity by excluding one outlying study (SMD ≤ −3.50) (9). One RCT compared three different treatment conditions (10 Hz tACS, 40 Hz tACS and sham). As recommended previously (19, 20), half of the participants were assigned to each active treatment arm (10 Hz-tACS or 40 Hz-tACS) to avoid inflating the number of participants in the sham group. Funnel plots or Egger’s test (21) were used to detect publication bias for the primary outcome. The significance level was set as alpha < 0.05, with two-sided tests.

### Assessment of study quality

Study quality was assessed using the Cochrane risk of bias (22) and Jadad scale (23) by the two independent authors (WZ and DBC). A Jadad score of ≥ 3 was defined as “high quality.” The two authors (WZ and DBC) independently assessed the overall quality levels of the primary and secondary outcomes using the Grading of Recommendations, Assessment, Development, and Evaluation (GRADE) system (24).

## Results

### Literature search

**FIGURE 1:**
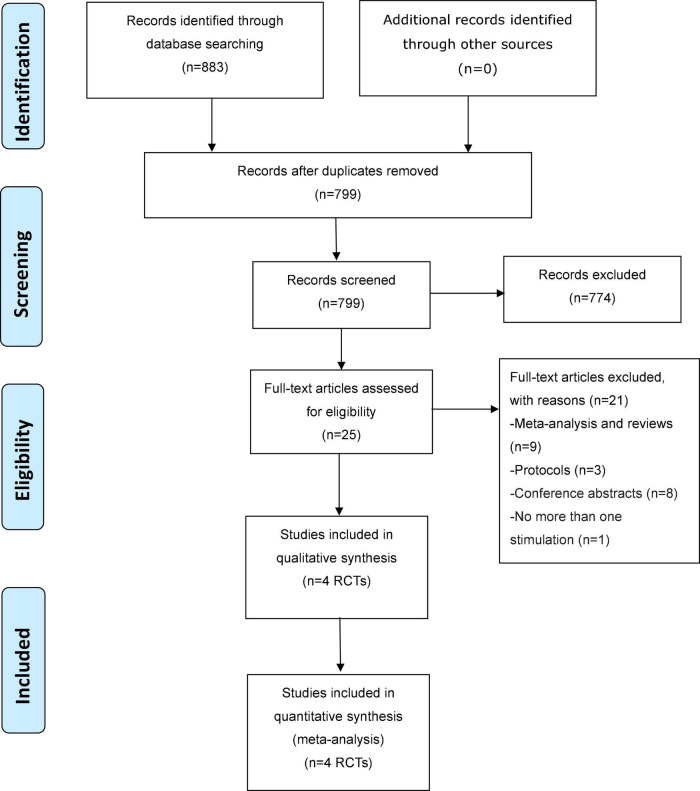
PRISMA flow diagram.

As depicted in Figure 1, a total of 883 potentially relevant articles were identified and screened in both Chinese (*n* = 592) and English (*n* = 291) databases and manual searches (*n* = 0). In total, 4 double-blind RCTs with 5 active treatment arms (8–11) met the study inclusion criteria.

### Patients and treatment characteristics

Across the 4 double-blind RCTs, 3 RCTs (3/4, 75%) were conducted in China, and one (1/4, 25%) was conducted in the USA (Table 1). Four RCTs with 5 active treatment arms (*n* = 224) compared adjunctive active tACS (*n* = 117) and sham tACS (*n* = 107). Almost all patients presented a diagnosis of MDD (*n* = 216, 96.4%), and only 8 (3.6%) were diagnosed with bipolar disorder. Their mean age was 38.8 (range = 32.1−41.1) years, and 20.1% (range = 13.3%−26.0%) of the patients were male. A 15 mA current intensity (77.5 Hz-tACS) was used in 3 RCTs (9–11), and one RCT with 2 active treatment arms applied a 1–2 mA protocol (10/40 Hz-tACS) (8).

**TABLE 1:** Summary of studies included in this meta-analysis.

References	Sample size (n)^a^	Design: -Blinding -Analyses -Setting (%)	Study duration (days)^b^	Participants: -Diagnosis (%) -Diagnostic criteria -Illness duration^c^ (yrs)	Mean age^c^ (yrs) (range)	Sex: male (%)	tACS therapeutic frequency and ADs dosages (mg/day); Number of patients (n)	-Montage anode (active/sham)^d^	-Intensity (mA) (anode electrode/cathode electrode)^e^ -Length (min) -Number of sessions (n/day)	Jadad score
Alexander et al. (8) (USA)	32	-DB -ITT -NR	5	-MDD (100) -DSM-5 -NR^f^	36.7 (18−65)	5 (15.6)	1. Sham tACS (active sham stimulation^g^) + ADs (NR^h^); *n* = 11 2. Active tACS (10 Hz) + ADs (NR^h^); *n* = 10 3. Active tACS (40 Hz) + ADs (NR^h^); *n* = 11	Anode: F3, F4 Cathode: Cz	−1/2 −40 −5 (1/day)	5
Luo et al. (9) (China)	62	-DB -OC -NR	20	-MDD (86.0) and BD (14.0) -DSM-5 -5.8	41.1 (18−65)	10 (17.5)	1. Sham tACS (no active stimulation) + ADs (NR^i^); *n* = 31 2. Active tACS (77.5 Hz) + ADs (NR^i^); *n* = 31	Fp1, Fpz, Fp2, Mastoid region of each side^j^	−15/15 −40 −20 (1/day)	5
Wang et al. (11) (China)	30	-DB -ITT -Outpatients	20	-First episode MDD (100) -DSM-IV -0.5	32.1 (18−65)	4 (13.3)	1. Sham tACS (no active stimulation) + drug naïve; *n* = 15 2. Active tACS (77.5 Hz) + drug naïve; *n* = 15	Fp1, Fpz, Fp2, Mastoid region of each side^j^	−15/15 −40 −20 (1/day)	5
Wang et al. (10) (China)	100	-DB -ITT -NR	20	-First episode MDD (100) -DSM-IV-TR -0.8	40.0 (18−65)	26 (26.0)	1. Sham tACS (no active stimulation) + drug naïve; *n* = 50 2. Active tACS (77.5 Hz) + drug naïve; *n* = 50	Fpz, Fp1, Fp2, Mastoid region of each side^j^	−15/15 −40 −20 (1/day)	5

^a^ Overall number of participants.

^b^ Study duration was defined as the time from the beginning to the end of tACS treatment.

^c^ Available data were extracted based on the mean baseline value of each included trial.

^d^ Electrode placement according to the International 10−20 system.

^e^ It is an average amplitude.

^f^ Symptom onset ranged from 1 year to more than 15 years.

^g^ Active sham stimulation included 20 s of ramp-in to 40 s of 10 Hz-tACS, with a ramp-out of 20 s, for a total of 80 s of stimulation.

^h^ Drug prescribed: SSRIs, SNRIs, tetracyclic, SARIs, aminoketone, lithium, benzodiazepine use as needed.

^i^ Antidepressant prescribed: SSRIs and benzodiazepine use as needed.

^j^ In the 10/20 international placement system, a 4.45 × 9.53 cm electrode was placed on the forehead corresponding to Fpz, Fp1 and Fp2. Two 3.18 × 3.81 cm electrodes were placed on the mastoid region of each side. ADs, antidepressants; BD, bipolar disorder; DB, double blind; DSM-IV, Diagnostic and Statistical Manual of Mental Disorders 4th edition; DSM-IV-TR, Diagnostic and Statistical Manual of Mental Disorders 4th edition, text revision; DSM-5,Diagnostic and Statistical Manual of Mental Disorders 5th edition; ITT, intent-to-treat; MDD, major depressive disorder; NR, not reported; OC, observed case; SARIs, 5-HT receptor antagonism and reuptake inhibitors; SNRIs, selective serotonin-norepinephrine reuptake inhibitors; SSRIs, selective serotonin reuptake inhibitors; tACS, transcranial alternating current stimulation; yrs, years.

### Assessment of the study quality

As shown in Supplementary Figure 1, the included 4 RCTs with an adequate double-blinded design mentioned “random” assignment with a specific description. Only one RCT (25%, 1/4) was rated as having an “unclear risk of bias” regarding allocation concealment (11). All included studies (Jadad score = 5) were rated as high quality (Table 1). According to the GRADE approach (Supplementary Table 2), the overall quality of the evidence for all seven meta-analytic outcomes was rated as “high” (5.9%, 1/17) or “moderate” (94.1%, 16/17).

### Primary outcome

**FIGURE 2:**
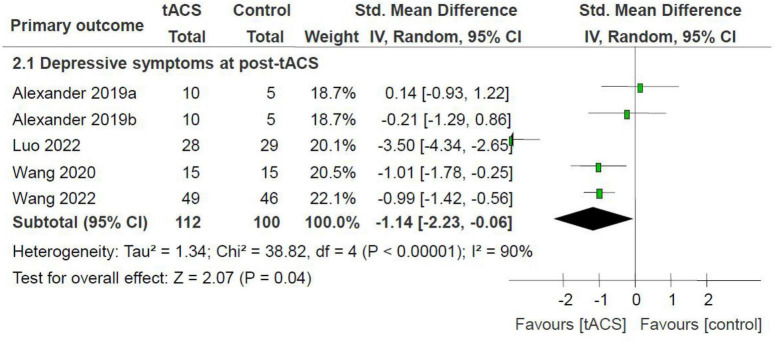
tACS for MDD: depressive symptoms at post-tACS as measured by the HAMD. HAMD, Hamilton Depression Rating scale; MDD, major depressive disorder; tACS, transcranial alternating current stimulation.

Meta-analysis of depressive symptoms at post-tACS as measured by the HAMD found an advantage of active tACS over sham tACS (4 RCTs with 5 active treatment arms, *n* = 212, SMD = −1.14, 95% CI: −2.23, −0.06; *I*^2^ = 90%, *P* = 0.04; Figure 2). Significance regarding depressive symptoms at post-tACS (*n* = 155, SMD = −0.69, 95% CI: −1.19, −0.18; *I*^2^ = 42%, *P* = 0.008) remained robust after removing one study (9) with an outlying effect size.

Similarly, meta-analysis of the depressive symptoms at the 4-week follow-up found an advantage of active tACS over sham tACS (*n* = 210, SMD = −1.07, 95% CI: −2.05, −0.08; *I*^2^ = 88%, *P* = 0.03; Table 2). Comparisons between the two groups in depressive symptoms at the 2 week follow-up (*n* = 29, SMD = −0.07, 95% CI: −0.83, −0.70; *I*^2^ = 0%, *P* = 0.86; Table 2) did not show significant differences.

**TABLE 2:** Secondary outcomes.

Variables	Study arms (subjects)	SMDs/RRs (95% CI)	*I*^2^ (%)	*P*
**Clinical efficacy**				
Depressive symptoms at 2 week follow-up	2 (29)	−0.07 (−0.83, 0.70)	0	0.86
Depressive symptoms at 4 week follow-up	5 (210)	−1.07 (−2.05, −0.08)	88	**0.03**
Study defined remission	4 (165)	2.07 (1.36, 3.14)	9	**0.0006**
Study defined response	4 (165)	1.31 (1.00, 1.73)	18	0.05
**Discontinuation rate**				
Discontinuation due to any reason	4 (205)	0.72 (0.23, 2.19)	0	0.56
**Adverse effects**				
Burning sensation	2 (32)	0.08 (−0.65, 0.81)	0	0.83
Headache	2 (32)	−0.17 (−0.91, 0.56)	0	0.64
Improved mood	2 (32)	0.55 (−0.20, 1.30)	0	0.15
Itching	2 (32)	0.31 (−0.43, 1.05)	0	0.42
Local redness	2 (32)	0.28 (−0.48, 1.04)	0	0.47
Neck pain	2 (32)	−0.09 (−0.82, 0.64)	0	0.81
Phosphene perception	2 (32)	1.19 (0.38, 2.00)	0	**0.004**
Scalp pain	2 (32)	0.21 (−0.52, 0.94)	0	0.57
Sleepiness	2 (32)	−0.03 (−0.76, 0.70)	0	0.94
Tingling	2 (32)	0.12 (−0.61, 0.85)	0	0.74
Trouble concentrating	2 (32)	−0.51 (−1.25, 0.23)	0	0.18

CI, confidence interval; RRs, risk ratios; SMDs, standardized mean differences. *p* < 0.05 is in bold.

### Study-defined remission and response

Active tACS was significantly superior to sham tACS regarding study-defined remission (60.7% versus 29.6%, *n* = 165, RR = 2.07, 95% CI: 1.36, 3.14, *I*^2^ = 9%, *P* = 0.0006; Table 2), but not regarding study-defined response (70.2% versus 52.1%, *n* = 165, RR = 1.31, 95% CI: 1.00, 1.73, *I*^2^ = 18%, *P* = 0.05; Table 2).

### Adverse effects and discontinuation rates

Compared with sham tACS, active tACS led to significantly increased phosphene perception (*n* = 32; SMD = 1.19; 95% CI: 0.38, 2.00; *I*^2^ = 0%, *P* = 0.004; Table 2). No significant differences were found with regard to other adverse effects (SMD = −0.51 to 0.55; 95% CI: −1.25, 1.30; *I*^2^ = 0%; *P* = 0.15 to 0.94; Table 2) or discontinuation due to any reason (*n* = 205; RR = 0.72; 95% CI: 0.23, 2.19; *I*^2^ = 0%, *P* = 0.56; Table 2) between the two groups.

### Publication bias

The funnel plot of the primary outcome presented a symmetrical distribution among the included RCTs (Supplementary Figure 2). As a minimum of 10 RCTs are required to run the Egger test, publication bias was not evaluated by conducting the Egger test (25).

## Discussion

In this meta-analysis of 4 double-blind RCTs with 5 active treatment arms (*n* = 224), active tACS for the treatment of MDD was significantly superior to sham tACS regarding the improvement of depressive symptoms at post-tACS and at the 4-week follow-up, as well as for study-defined remission. At the end of the treatment, 60.7% of patients showed remission and 70.2% showed response to active tACS compared with the corresponding figures of 29.6% and 52.1% for sham tACS. Although the administration of tACS resulted in a significantly higher rate of phosphenes, the rates of other adverse effects and discontinuation due to any reason were similar between the two groups.

The included RCTs, all with relatively small sample sizes (30 to 100), were published within the last 3 years, indicating that tACS for patients with MDD is a new, clinically important topic. The results of this meta-analysis suggest that tACS could provide a non-pharmacological alternative for the treatment of subjects suffering from MDD. For other NIBS techniques such as tDCS (26), the main objective is also to monitor their neurocognitive efficacy. For example, previous meta-analyses found that active tDCS was superior to sham tDCS in treating MDD (27) and improving attention/vigilance (26). Neurocognition is commonly affected in major mental disorders (28). However, in this meta-analysis, data on the neurocognitive efficacy of tACS for MDD were collected in only one RCT (25%, 1/4) (8). The neurocognitive efficacy of tACS for MDD should be further investigated.

The underlying mechanism of tACS in treating MDD maybe attribute to its feasibility in altering disturbed brain oscillations (7). The antidepressant effects of tACS for the treatment of MDD may depend on the different stimulation parameters, especially current and frequency (29). Among the included RCTs, tACS with an alternating current of 15 mA (75 Hz) was used in 3 RCTs (9–11), and tACS with a 2 mA current (10 Hz or 40 Hz) was used in one RCT with 2 active treatment arms (8). The current of 15 mA used in 3 out of 4 RCTs (75%, 3/4) (9–11) in this meta-analysis was higher than that used in previous reports (8, 30–32). Therefore, it remains unclear whether a smaller current or lower frequency can have dramatic antidepressant efficacy (10). Importantly, both alpha-tACS (8, 33) and gamma-tACS (6, 34) can effectively ameliorate depressive symptoms of MDD, suggesting that there are some specific gamma or alpha frequencies or other unknown frequencies that can significantly improve the symptoms of MDD (10).

In this meta-analysis, active tACS led to significantly increased phosphene perception when compared with sham tACS, which was reported in previous studies focusing on healthy subjects (35). A prior study (36) found that phosphene perception was most prominent at frequencies between 14 and 20 Hz-tACS. Numerous studies found that participants did not report phosphene perception at tACS with a higher frequency of ≥ 40 Hz (9–11, 37–39). In this meta-analysis, patients with MDD did not perceive phosphenes in three RCTs (75 Hz-tACS) (9–11) but did in Alexander et al.’s (8) study (10 Hz-tACS or 40 Hz-tACS). Similar to TMS (40) and tDCS (30), tACS used for treating MDD was safe and well tolerated.

This study has several limitations. First, the relatively small number of studies (4 RCTs covering 224 participants with MDD) precluded the evaluation of publication bias. Second, several previous studies reported that a 77.5 Hz pulsed alternating current appeared to produce the best stimulation of the antinociceptive system, eliciting maximal endorphin release and an analgesic effect (10, 41). However, the optimal parameters of tACS as a non-pharmacological alternative for the treatment of MDD should be further examined. Third, the meta-analytic results for the primary outcome were highly heterogeneous (*I*^2^ = 90), which was potentially attributed to heterogeneous samples, different study designs and different treatment protocols. However, the findings remained unchanged, and *I*^2^ decreased from 90 to 42% in the sensitivity analysis. Finally, only physically healthy patients suffering from MDD were recruited in included RCTs, limiting the generalizability of the findings.

In conclusion, tACS appeared to be modestly effective and safe in improving depressive symptoms for patients with MDD. More high-quality RCTs are warranted to explore the advantages of tACS as a therapeutic intervention for MDD.

## Data availability statement

The original contributions presented in this study are included in the article/Supplementary material, further inquiries can be directed to the corresponding author.

## Author contributions

WZ (first author), DB-C, and SN selected the studies, extracted the data, did the quality assessment of the included studies, and wrote the first version of manuscript. WZ (last author) reviewed all the data and helped solve disagreements. All authors have approved and contributed to finalize the final version of manuscript.
