# Supplementary material for: Adjunctive transcranial alternating current stimulation for patients with major depressive disorder: A systematic review and meta-analysis
Source: Front Psychiatry. 2023 Mar 22;14:1154354. doi: 10.3389/fpsyt.2023.1154354 (PMC10073427; doi:10.3389/fpsyt.2023.1154354)
Supplement: Supplementary file 1 [file Data_Sheet_1.docx]

Supplemental Table 1: PRISMA 2009 Checklist

| **Section/topic** | **#** | **Checklist item** | **Reported on page #** |
| --- | --- | --- | --- |
| **TITLE** | | |  |
| Title | 1 | Identify the report as a systematic review, meta-analysis, or both. | # 1 |
| **ABSTRACT** | | |  |
| Structured summary | 2 | Provide a structured summary including, as applicable: background; objectives; data sources; study eligibility criteria, participants, and interventions; study appraisal and synthesis methods; results; limitations; conclusions and implications of key findings; systematic review registration number. | # 3-4 |
| **INTRODUCTION** | | |  |
| Rationale | 3 | Describe the rationale for the review in the context of what is already known. | # 5 |
| Objectives | 4 | Provide an explicit statement of questions being addressed with reference to participants, interventions, comparisons, outcomes, and study design (PICOS). | # 5 |
| **METHODS** | | |  |
| Protocol and registration | 5 | Indicate if a review protocol exists, if and where it can be accessed (e.g., Web address), and, if available, provide registration information including registration number. | N/A |
| Eligibility criteria | 6 | Specify study characteristics (e.g., PICOS, length of follow-up) and report characteristics (e.g., years considered, language, publication status) used as criteria for eligibility, giving rationale. | # 6 |
| Information sources | 7 | Describe all information sources (e.g., databases with dates of coverage, contact with study authors to identify additional studies) in the search and date last searched. | # 6-7 |
| Search | 8 | Present full electronic search strategy for at least one database, including any limits used, such that it could be repeated. | # 6-7 |
| Study selection | 9 | State the process for selecting studies (i.e., screening, eligibility, included in systematic review, and, if applicable, included in the meta-analysis). | # 6-7 |

| **Section/topic** | **#** | **Checklist item** | **Reported on page #** |
| --- | --- | --- | --- |
| Data collection process | 10 | Describe method of data extraction from reports (e.g., piloted forms, independently, in duplicate) and any processes for obtaining and confirming data from investigators. | # 6-7 |
| Data items | 11 | List and define all variables for which data were sought (e.g., PICOS, funding sources) and any assumptions and simplifications made. | # 6 |
| Risk of bias in individual studies | 12 | Describe methods used for assessing risk of bias of individual studies (including specification of whether this was done at the study or outcome level), and how this information is to be used in any data synthesis. | # 8 |
| Summary measures | 13 | State the principal summary measures (e.g., risk ratio, difference in means). | # 7 |
| Synthesis of results | 14 | Describe the methods of handling data and combining results of studies, if done, including measures of consistency (e.g., I2) for each meta-analysis. | # 7 |
| Risk of bias across studies | 15 | Specify any assessment of risk of bias that may affect the cumulative evidence (e.g., publication bias, selective reporting within studies). | # 6-7 |
| Additional analyses | 16 | Describe methods of additional analyses (e.g., sensitivity or subgroup analyses, meta-regression), if done, indicating which were pre-specified. | # 6-7 |
| **RESULTS** | | |  |
| Study selection | 17 | Give numbers of studies screened, assessed for eligibility, and included in the review, with reasons for exclusions at each stage, ideally with a flow diagram. | # 8, Figure 1 |
| Study characteristics | 18 | For each study, present characteristics for which data were extracted (e.g., study size, PICOS, follow-up period) and provide the citations. | # 8-9, Table 1 |
| Risk of bias within studies | 19 | Present data on risk of bias of each study and, if available, any outcome level assessment (see item 12). | # 8-9, Supplemental Figure 1 |

| **Section/topic** | **#** | **Checklist item** | **Reported on page #** |
| --- | --- | --- | --- |
| Results of individual studies | 20 | For all outcomes considered (benefits or harms), present, for each study: (a) simple summary data for each intervention group (b) effect estimates and confidence intervals, ideally with a forest plot. | # 8-10, Table 1, Supplemental Figure 2 |
| Synthesis of results | 21 | Present results of each meta-analysis done, including confidence intervals and measures of consistency. | # 9-10, Figure 2, Table 2 |
| Risk of bias across studies | 22 | Present results of any assessment of risk of bias across studies (see Item 15). | #9-10, Supplemental Figure 1 |
| Additional analysis | 23 | Give results of additional analyses, if done (e.g., sensitivity or subgroup analyses, meta-regression [see Item 16]). | N/A |
| **DISCUSSION** | | |  |
| Summary of evidence | 24 | Summarize the main findings including the strength of evidence for each main outcome; consider their relevance to key groups (e.g., healthcare providers, users, and policy makers). | #10-11 |
| Limitations | 25 | Discuss limitations at study and outcome level (e.g., risk of bias), and at review-level (e.g., incomplete retrieval of identified research, reporting bias). | #11- 12 |
| Conclusions | 26 | Provide a general interpretation of the results in the context of other evidence, and implications for future research. | # 12 |
| **FUNDING** | | |  |
| Funding | 27 | Describe sources of funding for the systematic review and other support (e.g., supply of data); role of funders for the systematic review. | # 3 |

N/A: not applicable

*From:*  Moher D, Liberati A, Tetzlaff J, Altman DG, The PRISMA Group (2009). Preferred Reporting Items for Systematic Reviews and Meta-Analyses: The PRISMA Statement. PLoS Med 6(6): e1000097. doi:10.1371/journal.pmed1000097

For more information, visit: **www.prisma-statement.org**. Page 2 of 2

**Supplemental Table 2. GRADE Analyses: Adjunctive tACS for MDD**

| **Primary/***secondary* outcomes | **Study active arms (subjects)** | **Risk of bias** | **Inconsistency** | **Indirectness** | **Imprecision** | **Publication bias** | **Large effect** | **Overall quality of evidence^a^** |
| --- | --- | --- | --- | --- | --- | --- | --- | --- |
| **Depressive symptoms at post-tACS** | 4 (212) | No | Serious^b^ | No | Serious^c^ | Undetected | Large^d^ | +/+/+/-/; moderate |
| *Depressive symptoms at 2-week follow-up* | 2 (29) | No | No | No | Serious^c^ | Undetected | No | +/+/+/-/; moderate |
| *Depressive symptoms at 4-week follow-up* | 5 (210) | No | Serious^b^ | No | Serious^c^ | Undetected | Large^d^ | +/+/+/-/; moderate |
| *Study defined remission* | 4 (165) | No | No | No | Serious^c^ | Undetected | No | +/+/+/-/; moderate |
| *Study defined response* | 4 (165) | No | No | No | Serious^c^ | Undetected | No | +/+/+/-/; moderate |
| *Discontinuation due to any reason* | 4 (205) | No | No | No | Serious^c^ | Undetected | No | +/+/+/-/; moderate |
| *Burning sensation* | 2 (32) | No | No | No | Serious^c^ | Undetected | Large^d^ | +/+/+/+/; High |
| *Headache* | 2 (32) | No | No | No | Serious^c^ | Undetected | No | +/+/+/-/; moderate |
| *Improved mood* | 2 (32) | No | No | No | Serious^c^ | Undetected | No | +/+/+/-/; moderate |
| *Itching* | 2 (32) | No | No | No | Serious^c^ | Undetected | No | +/+/+/-/; moderate |
| *Local redness* | 2 (32) | No | No | No | Serious^c^ | Undetected | No | +/+/+/-/; moderate |
| *Neck pain* | 2 (32) | No | No | No | Serious^c^ | Undetected | No | +/+/+/-/; moderate |
| *Phosphene perception* | 2 (32) | No | No | No | Serious^c^ | Undetected | No | +/+/+/-/; moderate |
| *Scalp pain* | 2 (32) | No | No | No | Serious^c^ | Undetected | No | +/+/+/-/; moderate |
| *Sleepiness* | 2 (32) | No | No | No | Serious^c^ | Undetected | No | +/+/+/-/; moderate |
| *Tingling* | 2 (32) | No | No | No | Serious^c^ | Undetected | No | +/+/+/-/; moderate |
| *Trouble concentrating* | 2 (32) | No | No | No | Serious^c^ | Undetected | No | +/+/+/-/; moderate |
| Abbreviations: HAMD=Hamilton Depression Rating Scale; MDD=major depressive disorder; tACS=transcranial alternating current stimulation.  ^a^ GRADE Working Group grades of evidence: High quality=further research is very unlikely to change our confidence in the estimate of effect. Moderate quality=further research is likely to have an important impact on our confidence in the estimate of effect and could change the estimate. Low quality=further research is very likely to have an important impact on our confidence in the estimate of effect and is likely to change the estimate. Very low quality=we are very uncertain about the estimate.  ^b^ Meta-analytic results presented a serious inconsistency when I^2^ values were greater than 50% or P<0.1 in the Q statistics.  ^c^ For continuous outcomes, N<400. For dichotomous outcomes, N<300.  ^d^ Studies with large effects provided increased quality of evidence. Large effects=standard mean differences<-0.8 or >0.8 and risk ratios<0.5 or >2. | | | | | | | | |

**Supplemental Figure 1. Cochrane risk of bias**

|  | ***Random sequence generation (selection bias)*** | ***Allocation concealment (selection bias)*** | ***Blinding of participants and personnel*** | ***Blinding of outcome assessment (Symptom reduction, response)*** | ***Incomplete outcome data addressed (attrition bias)*** | ***Selective reporting (reporting bias)*** | ***Other sources of bias*** |
| --- | --- | --- | --- | --- | --- | --- | --- |
| Alexander et al., 2019 | **+** | **+** | **+** | **+** | **+** | **+** | **？** |
| Luo et al., 2022 | **+** | **+** | **+** | **+** | **+** | **+** | **？** |
| Wang et al., 2020 | **+** | **？** | **+** | **+** | **+** | **+** | **？** |
| Wang et al., 2022 | **+** | **+** | **+** | **+** | **+** | **+** | **？** |

+: Low risk of bias, -: High risk of bias; ?: Unclear risk of bias

**Supplemental Figure 2. funnel plot for primary outcome**


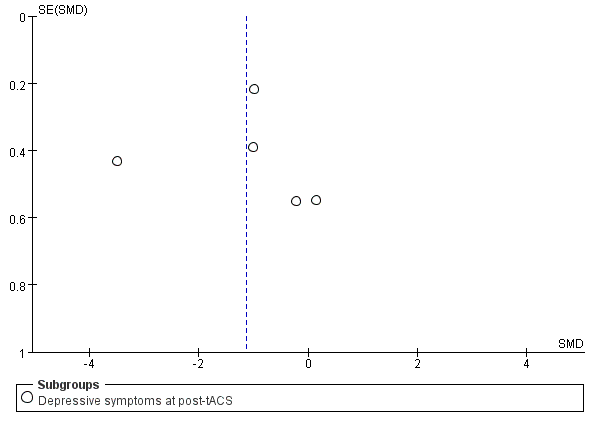


**Supplemental Text: data searches**

The databases searched included:

- Pubmed
- Cochrane Library.
- PsycINFO
- EMBASE
- Chinese National Knowledge Infrastructure
- Wanfang database

**Search detail:**

**Pubmed**

Via https://pubmed.ncbi.nlm.nih.gov/

Searched on: 1 April 2022

Records retrieved: 53

Search term:

("transcranial alternating current stimulation" OR tACS) AND ("depression"[MeSH] OR depression OR depressive OR depressed OR melancholia)

**Cochrane Library**

via https://www.cochranelibrary.com/advanced-search

Searched on: 1 April 2022

Records retrieved: 60

Search term:

(transcranial alternating current stimulation OR tACS) AND (depression OR depressive OR depressed OR melancholia)

**PsycINFO**

via Ovid http://ovidsp.ovid.com/

Searched on: 1 April 2022

Records retrieved: 34

Search term:

(transcranial alternating current stimulation OR tACS) AND (depression OR depressive OR depressed OR melancholia)

**EMBASE**

via https://www.embase.com/

Searched on: 1 April 2022

Records retrieved: 144

Search term:

(‘transcranial alternating current stimulation’ OR ‘tACS’) AND (‘depression’/exp OR ‘depression’ OR ‘depressive’ OR ‘depressed’ OR ‘melancholia’)

**Chinese National Knowledge Infrastructure**

via https://www.cnki.net/

Searched on: 1 April 2022

Records retrieved: 5

Search term:

SU=(‘情感障碍’ + ‘心境障碍’ + ‘抑郁症’ + ‘抑郁发作’ + ‘抑郁’ + ‘忧郁’ + ‘忧郁症’) * (‘经颅交流电刺激’ + ‘经颅电刺激’ + ‘tACS’)

**Wanfang database**

via https://www.wanfangdata.com.cn/

Searched on: 1 April 2022

Records retrieved: 587

Search term:

(情感障碍 OR 心境障碍 OR 抑郁症 OR 抑郁发作 OR 抑郁 OR 忧郁 OR 忧郁症) AND (经颅交流电刺激 OR经颅电刺激 OR tACS)
